# Teacher Evaluations of Executive Functioning in Schoolchildren Aged 9–12 and the Influence of Age, Sex, Level of Parental Education

**DOI:** 10.3389/fpsyg.2017.00481

**Published:** 2017-04-03

**Authors:** Marleen A. J. van Tetering, Jelle Jolles

**Affiliations:** Centre for Brain and Learning, Faculty of Behavioral and Movement Sciences, Vrije Universiteit AmsterdamAmsterdam, Netherlands

**Keywords:** executive functions, developmental psychology, young adolescence, late childhood, sex differences, level of parental education

## Abstract

Executive functions (EFs) develop over the period of early childhood and adolescence up until young adulthood. Individual children differ substantially in the pace at which their EFs develop, and characteristics such as sex and the level of parental education (LPE) are thought to contribute to these differences. In the present study, we assessed age-related changes in EFs as *perceived and evaluated by teachers and parents* as well as the influence of sex and LPE on their evaluations. We used a newly developed observer-report questionnaire, the Amsterdam Executive Function Inventory (AEFI). The AEFI assesses three important components of the executive aspects of daily life behavior in 13 questions: Attention; Self-control and Self-monitoring; and Planning and Initiative taking. Teachers and parents evaluated these aspects of executive functioning in 186 schoolchildren in grades 3–6 (age: 9–12 years). Age effects within grades and differences in social economic status between the four participating schools were controlled. Results showed a significant increase in teacher-perceived EFs from third to fourth grades and from fifth to sixth grades. This development was influenced both by the sex of the child and by the LPE. As perceived by teachers, the component self-control and self-monitoring was higher for girls than for boys, and planning abilities were higher for children from families with a higher LPE. Additional analyses showed that there is a systematic and statistically significant difference between the evaluations of the teachers and that of parents. Parents reported higher scores for planning, whereas teachers reported higher scores for self-control and self-monitoring. Evaluations by parents and teachers were different for girls, but not for boys. These findings are important because they imply that the development of EFs as perceived by parents and teachers is influenced by child-related factors. Second, there are clear differences in evaluations between teachers and parents. The AEFI appears to be a tool that is easily used by parents and teachers and shows potential for monitoring the development of EFs as perceived by significant others during young adolescence.

## Introduction

The so-called ‘executive functions’ (EFs) play an important role in the development of children and adolescents (Beauchamp and Anderson, 2010; Diamond, 2013), and have recently been mentioned as possible determinants in educational success (Best et al., 2011; Kautz et al., 2014; Titz and Karbach, 2014). Children who perform well in the domain of EFs early in life achieve better academically at a later age (Best et al., 2011; Diamond, 2013; Kautz et al., 2014; Rabiner et al., 2016). Particular EFs start to develop in early childhood and continue to develop and mature with experience until at least emerging adulthood (i.e., age 18–23) (Giedd et al., 1999; Gogtay et al., 2004; Giedd and Rapoport, 2010; Taylor et al., 2013). Major changes in the EFs take place in the period of late childhood and young adolescence – the period between 8 and 12 years – and both the sex of the child and the level of education of their parents (LPE) may affect this development (Lenroot and Giedd, 2010; Hackman et al., 2010; Lemos et al., 2011; Lemos et al., 2011; Diamond, 2013; Hyde, 2014; Miller and Halpern, 2014; Rindermann and Baumeister, 2015). The influences of age, sex and LPE are investigated in the present study.

Between the ages of 8–12, a child undergoes major biological, behavioral and social changes (Leshem, 2016). Puberty starts within this period and substantial changes take place in the endocrine system and in the brain (Lenroot and Giedd, 2010; Diamond, 2013; Jolles, 2016). Brain systems mature and children become better able to cope with the changing demands that their social context – parents, family, neighborhood, school and significant others – places upon them. The brain learns to distinguish relevant information from the enormous amount of sensory irrelevant information that the environment offers. This enables a child to concentrate and to stay focused for a longer period of time (Diamond, 2013; Jolles, 2016). Children become less impulsive over the years by learning to reflect on their own behavior before acting. This enables a child to choose for the best behavioral alternative (Diamond, 2013; Lawrence et al., 2015; Jolles, 2016; Leshem, 2016). Children learn to prioritize, and to anticipate on future events. They learn to make a planning to accomplish their future goals, to evaluate on the effectiveness of their planning and to adjust it to become more effective. Children get experience with planning on the short- and on the long term, and to keep in mind the consequences of their behavior for other individuals. Attention, self-regulation and planning are three core EFs that enable the developing child to become more skilled, get experience, and to adapt to the changing situations in their everyday life (Lowe and Cook, 2003; Casey et al., 2010; McCloskey and Perkins, 2012; Diamond, 2013; Leshem, 2016).

The present study aimed to provide insight into the development of EFs as evaluated by teachers and parents in relation to two child-related factors, namely sex and LPE. An important characteristic of the study was the evaluation of *evaluations* made by teachers and parents with respect to the EFs of the child. The choice for observer-reports was made because only a fraction of the children could be expected to be able to judge their own behavior in a more or less objective way. This is because self-reflection is one of the EFs that is developing actively during early childhood and adolescence (Diamond, 2013; Weil et al., 2013). The teachers and parents were asked to assess those aspects of daily life behavior that have particular relevance for scholastic performance and daily life functioning, namely: attention, planning and initiative taking, and self-control and self-monitoring (Gioia et al., 2000; Isquith et al., 2004; Van der Elst et al., 2012; Diamond, 2013).

With respect to the biological mechanisms underlying the changes in EFs that occur between the ages of 9–12 years, scientific evidence suggests that important milestones are reached in the maturation of brain networks. During this period, major structures in the prefrontal areas are connected with structures in subcortical areas, brainstem, and posterior cortex, with the development of networks as a result (Huizinga et al., 2006; Lenroot and Giedd, 2010; Leshem, 2016). These networks mature further over the long period of adolescence until emerging adulthood (i.e., age 18–23 years) (Steinberg and Morris, 2001; Casey et al., 2008; Giedd and Rapoport, 2010; Steinberg, 2014; Baars et al., 2015). The brain activity connected to these brain networks is responsible for the development of EFs, and thus for planning, regulating, evaluating and controlling behavior and thoughts in relation to situational demands (Zimmerman, 2000; Zimmerman and Schunk, 2001; Shaw et al., 2008; Beauchamp and Anderson, 2010; Giedd and Rapoport, 2010). The development of EFs matches the stages of brain maturation (Huizinga et al., 2006; Best et al., 2011; Baars et al., 2015), and a progressive improvement in EFs linked to age has been observed (Ardila et al., 2005; Huizinga et al., 2006).

Yet, already at the end of primary school there are substantial individual differences in the pace at which children develop physically, but also in their learning motivation and academic achievements. Some children are characterized by superior school grades and by an interest in knowledge acquisition, whereas others are playful and have less interest in cognitive learning (Vecchione et al., 2014). It is probable that these individual differences are related to the pace at which EFs develop, and thus to both biological and psychosocial factors (Ardila et al., 2005; Hackman et al., 2010; Lenroot and Giedd, 2011; Dekker et al., 2013; Diamond, 2013; Miller and Halpern, 2014; Noble et al., 2015). The biological factors are affected by physical and brain maturation as well as by factors such as the sex of the child (Lenroot and Giedd, 2011; Dekker et al., 2013; Diamond, 2013; Miller and Halpern, 2014). Psychosocial factors are related to social background, the physical environment in which the child develops, and the LPE (Ardila et al., 2005; Hackman et al., 2010; Miller and Halpern, 2014; Noble et al., 2015). Hence, the present study aimed to improve our understanding of the influence of two child-related factors, namely sex and LPE, on the development of teacher and parent-perceived EFs.

The first child-related factor we examined on the development of perceived EFs is sex differences. The majority of boys and the majority of girls are found to differ in the pace and time path with respect to the development of their EFs (Lenroot and Giedd, 2010; Diamond, 2013; Hyde, 2014; Miller and Halpern, 2014; Rindermann and Baumeister, 2015). Girls appear to outperform boys on verbal fluency tasks and boys have lower levels of inhibitory control than girls (Berlin and Bohlin, 2002; Miller and Halpern, 2014). Moreover, the incidence of problems in the domain of EFs decreases as children grow older. This decrease was greater for girls than for boys (Gioia et al., 2000; Huizinga and Smidts, 2010). Recently, Miller and Halpern (2014), in their authoritative review on sex differences in cognitive abilities, stated that earlier literature needed to be reexamined. This is due to new findings about trends over time, infant cognition, sex hormones, brain differences, culture and stereotypes (Miller and Halpern, 2014). With respect to brain differences, longitudinal studies have shown sex differences in the trajectory of brain development, with females reaching peak values of brain volumes earlier than males (Lenroot and Giedd, 2011; Miller and Halpern, 2014). This indicates that there are sex differences in the pace and/or trajectory at which EFs develop.

The second child-related factor we examined is the LPE (Hackman et al., 2010; Lemos et al., 2011). The LPE is regarded to be an ‘approximation’ or ‘proxy’ – a term used in epidemiology – of the intellectual and ‘growth-promoting’ climate within a family. LPE is a proxy for the complexity of the language used, the nature and the number of books read, and the level of ambition that parents have for their developing child (Ardila et al., 2005; Meijs et al., 2009; Evans et al., 2010). It has also been related to children’s school attendance and general cognitive development (Ganzach, 2000; Carr and Pike, 2012; Kautz et al., 2014). We have shown earlier that parental education and occupation influence problem-solving behavior and the attention of children (Hurks et al., 2006; Meijs et al., 2009).

Given the literature on the development of EFs and brain maturation, as well as the research findings on possible boy-girl differences and the influence of LPE, the following four research questions were addressed in this study: (1) Is there a change in teacher-perceived EFs in the period between grades 3–6, (ages 9–12 years)? (2) Does sex play a role in the development of the teacher-perceived EFs? (3) Does LPE play a role in the development of the teacher-perceived EFs? (4) Do teachers and parents differ in their evaluations of the development of EFs in their student/child? The last question was informed by the consideration that the evaluations of parents are probably influenced by the ambitions that they have for the future of their child. The teachers’ evaluations, on the other hand, are probably based on their experiences with the child in the classroom, on comparisons with other children, and on the child’s behavior in the school context. It is to be expected that the teachers’ evaluations will be influenced by their perception of the learning attitude of the pupil and by disruptive behavior. Knowledge with respect to differences in evaluations of teachers and parents is important for several reasons. First, academic, behavioral and social-emotional skills are the cumulative product of earlier learning experiences within multiple overlapping environments, such as at home and at school (Sheridan et al., 2017). Knowledge about differences between evaluations of parents and teachers gives a more appropriate overview of EFs of the child in different environments (Sheridan et al., 2017). Evaluations of EFs are an indication of the expectations of teachers and parents. These expectations are important for how teachers and parents behave toward the child. For instance, teachers frequently reported drawing on their beliefs about a child’s abilities when determining how to respond to children’s interaction (Summers et al., 2017). Teachers may give more challenging assignments to children if they believe that they are able to pay attention for a longer period of time and to plan their schoolwork properly (Summers et al., 2017). Parents observe the behavior and cognition of their child in the home setting. High expectations of parents about the self-control of their child will probably give rise to assignments with higher responsibilities (Shumow and Miller, 2001). Evaluations of teachers may thus be related to performances at school, while evaluations of parents are related to performances at home. Second, comprehension of parents and teachers about possible differences in their evaluations may be beneficial for the development of a more appropriate and effective educational strategy to a child’s individual needs. Improvement in the parent–teacher relationship was linked to a decrease in teacher-reported behavioral problems, and an increase in appropriate task related behavior (Sheridan et al., 2017). Third, researchers and clinician frequently use observational reports to evaluate EFs of children (e.g., Nilsen et al., 2017; Taveras et al., 2017). Differences in evaluations of teachers and parents indicate that it is important to be aware who evaluated the EFs.

In this study, EFs as perceived by teachers and parents were measured with the aid of a newly developed, validated, observer-report questionnaire, the Amsterdam Executive Function Inventory (AEFI). In this study, this instrument has been developed to enable a rapid and reliable evaluation of EFs as evaluated by a significant other, such as the teacher and the parent of the child (Van der Elst et al., 2012). Since 2000, there have been many studies in which observer-reported EFs have been examined. Many of these studies have used the BRIEF (Gioia et al., 2000; Huizinga and Smidts, 2010; Skogan et al., 2016). Observer-report questionnaires are intended to measures the semi-quantitative assessment of child’s behavior as evaluated by others (Lezak, 2004). However, they do not measure actual *cognitive performance*, for which dedicated neurocognitive tests are the instrument of choice. Accordingly, the observer-report questionnaires do measurements in another behavioral domain.

We used an observer-report questionnaire for the present study because we were interested in the development of behavior as perceived and evaluated by teachers in classrooms and by parents at home. The AEFI focuses on three aspects of EFs, namely: (1) Attention; (2) Planning and Initiative taking; and (3) Self-control and Self-monitoring. These aspects of EFs have also been described in many earlier papers (Gioia et al., 2000; Van der Elst et al., 2012; Diamond, 2013).

We conducted a quasi-experimental study in four mainstream primary schools, comprising 186 individuals in grades 3–6 (aged 9–12 year). The study was designed to yield a study population that was homogenous. Accordingly, age, sex and earlier life and learning experiences as related to the LPE of the child were controlled for. Additionally, individuals who skipped or repeated a class were deleted from the study in order to control for possible age effects within grades. The selected schools were chosen from the same population of primary schools and were equivalent with respect to the general educational philosophy behind the school and educational plan.

## Materials and Methods

### Participants and Procedure

The study was part of a cross-sectional research program into the determinants of learning performance and neurocognitive development during late childhood and young adolescence (i.e., grades 3 to grade 6). Participants were recruited from four mainstream primary schools in a rural area near Amsterdam (the Netherlands). The schools had the same board and they were chosen to provide roughly equivalent numbers of children from low, medium and higher social economic status (SES). Thus, at the first school the majority of children were from low SES families, while the second and third schools contained mainly children from moderate SES families, and the fourth school contained mainly children from high SES families. Children with different ethnicities were randomly distributed over the grades. Parents or caregivers (referred to as caregivers in the rest of the paper) received an information letter about the study and could withdraw their child from participation by signing and returning an objection form. Participation in this study was voluntary. All caregivers were informed that no personalized data would be used in the analyses and no personalized results would be obtained, since all data were assembled on group level.

One of the caregivers indicated the highest level of education attained in their family on a commonly used Dutch educational rating scale ranging from primary school (1) to post-university degree (9) (Bie, 1987). The system is similar to the International Standard Classification of Education (Singh, 2010). Two groups were created. The first group was classified as having a relatively high LPE (higher than vocational training), and the second group was classified as having relatively moderate-to-low LPE (vocational training or lower). After approval by the caregivers, one of the parents was invited to fill out a hardcopy of the observer questionnaire related to the EFs of their child and the teachers received login details per e-mail to fill out the same observer questionnaire for every child individually (∼8 min per questionnaire).

Of the 310 children who participated, 186 children (92 boys, 94 girls) were selected for the current study. This selection was based upon the following exclusion criteria: (a) repetition or skipping of a grade (*n* = 86); (b) missing data on the questionnaire used by teachers (*n* = 11); and (c) unreliable data due to technical problems (*n* = 6). Information about the LPE was missing for 25 children. For these children, mean LPE was imputed by the mean LPE of the grade from the school of the child. In addition, equal gender ratios between grades were created to control for sex effects within grades. This was done because the four grades showed substantial differences in the ratio between boys and girls. Boys and girls were randomly paired per school based on their age. As a result, 12 boys within grade 5 and nine boys within grade 6 were randomly excluded from the study. The age of the participating children ranged from 8.5 (grade 3) to 12.5 years (grade 6). The numbers of children were approximately the same per grade. The demographics and characteristics of the 186 children are presented in **Table 1**. For all variables, normality assumptions were checked (i.e., 1; skewness < 3, kurtosis < 10; Kline, 2005).

**Table 1:** Demographics and characteristics of 186 children.

Grade	3	4	5	6
N	49	49	47	41
Mean Age (*SD*)	8.92 (0.30)	10.1 (0.26)	11.07 (0.27)	11.94 (0.31)
Boys/Girls (N)	24/25	24/25	23/24	21/20
High/moderate to low LPE (N)	25/24	22/27	25/22	20/21

With respect to data for use in answering research question 3, the data of 24 children were missing on the questionnaire used by parents. These children were excluded from analyses in which evaluations by teachers were compared with evaluations by parents. Accordingly, 162 children were used for answering research question 3.

### Measures

#### Amsterdam Executive Functioning Inventory

The Amsterdam Executive Functioning Inventory (AEFI) (Van der Elst et al., 2012) was originally developed to measure EFs by means of a short self-report questionnaire. It consists of 13 items, representing three dimensions of EFs, namely the level of: (1) Attention (e.g., three items); (2) Planning and Initiative taking (e.g., five items); and, (3) Self-control and Self-monitoring (e.g., five items). The 13 questions in the original version of the AEFI were identical to those used in the present study. For the current study we used an observer-report version of the AEFI and there were slight differences in some examples given to explain the questions, in order to make the questions age appropriate (see Table 5 in Appendix). Teachers and parents were asked to indicate how well each item described the child by endorsing one of three responses on a 3-point Likert scale: ‘1 = not true,’ ‘2 = partly true,’ ‘3 = true.’ Items 1,4,5,6,7,8,11,12, and 13 were reverse coded, and total score of all items was calculated so that higher scores were indicative of better perceived EFs. In our study, the AEFI did not intend to relate perceived EFs to any cognitive measure per subject, and the instrument does not have the ambition to be useful for clinical purposes.

The internal consistency and the reliability the AEFI as observer-report were assessed, since the AEFI has originally been developed as self-report questionnaire. Validity of the AEFI was evaluated previously in a large study of adolescents aged 15–18 years and has been reported to be adequate (Van der Elst et al., 2012). Cronbach’s alpha coefficients (which should be ≥ 0.6–0.7; Dekovic et al., 1991; Holden et al., 1991; Clark and Watson, 1995) of the scales attention, planning and initiative taking and self-control and self-monitoring have been reported to be adequate (0.80, 0.81, 0.74 and 0.59, 0.60, 0.65 for teachers and parents respectively). In addition, the corrected item-scale correlations (i.e., the correlations between items and scale scores that did not include the items being evaluated), were calculated which should be ≥ 0.30 (Ferketich, 1991). Taking into account the Cronbach’s alpha coefficients and the corrected-item scale values, we conclude that the reliability of the AEFI used as observer report is adequate. For shorter scales, the corrected item-scale values provide a better index of internal consistency and reliability than Cronbach’s alpha, because Cronbach’s alpha values are not only a function of the height of the inter-correlations between the items of a scale, but also a function of the number of items on that scale (Clark and Watson, 1995). The corrected item-scale values separately for teachers and parents are reported in the Appendix of this article.

### Statistical Analyses

Age group differences, sex differences, and LPE differences were investigated by three separate one-way analyses of variance (ANOVA’s). The dependent variables included the means of the three AEFI scales (i.e., attention, planning and self-control), and the total score on these three scales as proxy of EFs in general. Grade (grades 3–6), sex (boy or girl), and LPE (high or low) were included as independent variables.

As a final test, paired samples *t*-tests were performed to investigate differences in evaluations between parents and teachers. The dependent variables include the three AEFI scales and the total score. *Post hoc* analyses were performed to investigate whether evaluations of teachers and parents were influenced by the sex of a child.

Modified Hochberg correction was used to correct for Type-1 errors due to multiple testing (Rom, 2013). Accordingly, *p*-values equal or smaller than 0.03 were considered statistically significant. All analyses were performed using SPSS version 23.

## Results

### Differences in Perceived EFs between Grades

Results of the one-way ANOVA revealed a significant main effect for grade on the attention scale [*F*(3,182) = 9.27, *p* = 0.00], on the scale self-control and self-monitoring [*F*(3,182) = 8.30, *p* = 0.00], and on the total AEFI score [*F*(3,182) = 5.80, *p* = 0.00]. There were significant increases in means between grades 3 and 4 (attention, *p* = 0.03; self-control and self-monitoring, *p* = 0.00; total AEFI, *p* = 0.02), and between grades 5 and 6 (attention, *p* = 0.00; self-control and self-monitoring, *p* = 0.00; total AEFI, *p* = 0.03). No significant increases with age were found on the scale planning (**Table 2**).

**Table 2:** Grade-related changes on the AEFI scales.

Grade					
	3	4	5	6	*p*-value
Total AEFI score	15.24 (6.41)	17.90 (4.93)	17.53 (6.37)	20.17 (4.24)	0.00^∗^
*AEFI Scales* Attention	3.02 (1.97)	3.82 (1.67)	3.68 (1.91)	4.95 (1.28)	0.00^∗^
Planning and initiative taking	5.43 (3.10)	6.02 (2.38)	6.64 (2.89)	6.32 (2.83)	0.19
Self-control & self-monitoring	6.80 (2.40)	8.06 (2.00)	7.21 (2.67)	8.90 (1.20)	0.00^∗^

*^∗^*p* ≤ 0.03.*

### Sex Differences in Perceived EFs

One-way ANOVA revealed a significant main effect for sex on the scale self-control and self-monitoring [*F*(1,184) = 6.18, *p* = 0.01]. Mean self-control and self-monitoring was evaluated higher for girls (mean = 8.11, *SD* = 2.08) than for boys (mean = 7.28, *SD* = 2.43). No sex differences were reported on the other two scales or on the total AEFI score (**Table 3**).

**Table 3:** Sex differences on the AEFI scales.

Sex				
**Scale**	**Boys**	**Girls**	**d**	***p*-value**
Total AEFI score	17.18 (5.77)	18.02 (5.88)	0.45	0.33
*AEFI Scales* Attention	3.67 (1.86)	3.97 (1.85)	0.16	0.28
Planning and initiative taking	6.23 (2.85)	5.95 (2.80)	0.10	0.50
Self-control and self-monitoring	7.28 (2.42)	8.11 (2.08)	0.37	0.01^∗^

*^∗^*p* ≤ 0.03.*

### A Comparison of Children from High and Low-to-Moderate LPE Families

Results of the one-way ANOVA revealed significant differences in mean score on the scale planning (mean difference = 0.96, *p* = 0.02). Mean was higher for children from high LPE families compared to children from low-to-moderate LPE families (**Table 4**).

**Table 4:** Level of parental education (LPE) differences on the AEFI scales.

LPE				
**Scale**	**Low – Moderate**	**High**	**d**	***p*-value**
Total AEFI score	17.74 (5.87)	18.46 (5.68)	0.13	0.04
*AEFI Scales* Attention	3.64 (1.88)	4.00 (1.83)	0.19	0.19
Planning and initiative taking	5.60 (2.92)	6.56 (2.65)	0.34	0.02^∗^
Self-control and self-monitoring	7.50 (2.33)	7.89 (2.25)	0.17	0.24

*^∗^*p* ≤ 0.03.*

Additional analyses in which the interaction between age and sex has been investigated revealed that there were no significant interactions on any of the AEFI scales [attention: *F*(3,178) = 0.262, *p* = 0.85; planning: *F*(3,178) = 1.38, *p* = 0.25; self-control and self-monitoring: *F*(3,178) = 0.15, *p* = 0.93; total AEFI: *F*(3,178) = 0.383, *p* = 0.77].

### Differences between Teachers’ and Parents’ Evaluations

Paired samples *t*-tests revealed significant differences in means between teachers and parents on the scales planning [*t*(161) = 3.02, *p* = 0.00] and self-control [*t*(161) = -3.11, *p* = 0.00]. Specifically, parents reported a higher mean on the scale planning (mean difference = 0.69), and teachers reported a higher mean for self-control and self-monitoring (mean difference = 0.58).

*Post hoc* analyses revealed that the evaluations of parents and teachers on the self-control and self-monitoring scale and on the planning scale were different for girls, but not for boys. For girls, the teachers’ mean evaluation was higher than that of parents on the self-control and self-monitoring scale (mean difference = 1.05, *p* = 0.00), and parents’ score was higher than teachers’ score on the planning scale (mean difference = 0.79, *p* = 0.02).

Finally, *post hoc* one-way ANOVA revealed that parents did not report significant differences between girls and boys on any of the scales.

## Discussion

The results of this large-scale cross-sectional study show that teachers observed an improvement in their pupils with regard to the components attention and self-control and self-monitoring, in the years between late childhood to young adolescence (i.e., 8–12 years). Moreover, teachers’ evaluations were influenced by the sex of the child and the LPE. These findings imply that these child characteristics may be determinants of differences in the pace at which EFs as perceived by teachers develop.

First of all, our results specifically indicate a significant age-related increase in the teacher-perceived EFs from the third to fourth grades and from the fifth to sixth grades. Secondly, development in EFs as perceived by teachers was influenced by the sex of the child, since teacher-perceived self-control and self-monitoring were higher for girls than for boys. Thirdly, teachers evaluated planning and initiative taking higher for children from families with a higher LPE. No significant interactions were found between age and sex. Finally, our findings revealed the important finding that there were differences between teachers’ and parents’ evaluations. Parents’ evaluations were higher for planning and initiative taking, while teachers reported higher scores for self-control and self-monitoring. These differences in evaluations were only reported for girls, and not for boys.

The results of this study provide us with important new insights into the determinants of differences in the pace at which EFs, as perceived by teachers, develop. With respect to sex differences, particular longitudinal studies have shown a time lag in brain development in boys (Lenroot et al., 2007; Giedd, 2008; Lenroot and Giedd, 2011). Higher self-control and self-monitoring for girls, as reported by teachers in this study, might be explained by the later onset of maturation in brain areas related to these important elements within the EFs umbrella in boys (De Bellis et al., 2001; Lenroot et al., 2007; Giedd, 2008; Lenroot and Giedd, 2011; Baars et al., 2015). Lower self-control and self-monitoring skills for boys as reported by teachers may indicate that assignments and guidance at primary school level should be more structured than for girls.

Another child-related factor that appears to have a substantial effect on the development of EFs as perceived by teachers is LPE. The LPE is regarded to be a ‘proxy’ of the intellectual and emotional climate within a family. This climate affects the support that the child gets from the parents, the complexity of the language used in the family, the books read, the availability of playing materials, the level of ambition the parents have for their developing child, as well as school attendance and general cognitive development (Ganzach, 2000; Evans et al., 2010; Carr and Pike, 2012; Kautz et al., 2014; Rindermann and Baumeister, 2015). Results of the present study are a strong support for our view that these factors positively stimulate the development of EFs as perceived by teachers, and thereby contribute to better school performance and a more positive attitude of the child toward learning. Findings of our study suggest that the development of educational programs for boys and for children from lower LPE and income families could provide these children with materials that would improve their cognitive and psychological development.

Another important finding of this study indicates differences in the evaluations by teachers and parents. Our hypothesis that parents and teachers differ in their evaluations was confirmed only with regard to girls. Hence, parents were more positive about girls’ planning abilities and initiative taking, and teachers were more positive about girls’ self-control and self-monitoring. Results of the present study contribute to create a better understanding of parents and teachers about possible differences in their evaluations. This is beneficial for the development of a more appropriate educational strategy to a child’s individual needs in order to take the strengths and weaknesses of a child into account. Furthermore, it is important for researchers and clinicians to be aware who evaluated the EFs when interpreting the results of an observer-report questionnaire. Differences between parents and teachers in their evaluations may be related to differences in their expectations, and to the experiences and emotions involved in their evaluations. An explanation for this could be that teachers’ evaluations are usually mainly based on objective academic achievements and on their quite global evaluation of the behavior of the child in the class. In this respect, teachers compare their students with students they have encountered in earlier years, while parents know their children much better and evaluate them on the basis of other criteria than those used by the teacher.

Moreover, it is of interest that evaluations of parents and teachers were influenced by the sex of the child. The higher mean for self-control and self-monitoring for girls in the case of teachers compared to parents might be related to the fact that teachers have more experience with the different behavior of boys and girls in their class. On the other hand, higher planning skills in the case of girls as reported by parents may be related to differences in the environmental setting in which parents and teachers observe children’s behavior. At school, given instructions are highly structured. For example, children are told to do one task from their mathematics book. For such tasks, high levels of self-control are necessary, and the students rely less on planning abilities. Parents observe children at home, however, and here instructions are more open for the child’s own interpretation, such as going to the supermarket to get some groceries. Such tasks rely more on planning abilities, and parents observe how their children grow in such skills and are able to take more responsibility over the years. The behavior of girls is in line with the behavioral expectations believed to be important for school (Kautz et al., 2014). Therefore, their behavior is easy to evaluate by teachers and parents, and differences in evaluations between teachers and parents are a result of differences in the environmental settings in which they observe their children’s behavior. In contrast, the behavior of boys may not match behavioral expectations which are believed to be important for school, which makes the evaluation of boys’ behavior more complicated and less accurate (Miller and Halpern, 2014; Jolles and Keizer, 2015). For instance, most boys are more playful then girls at the age 8–12 (Miller and Halpern, 2014; Jolles and Keizer, 2015; Jolles, 2016). Parents and teachers may not evaluate planning abilities of boys while playing (e.g., building a tent) as an important skill for school. As a result, evaluations of teachers and parents for EFs of boys are lower in general, and they do not systematically differ between teachers and parents. It is important that teachers and parents understand why their assessments of a child’s functioning might differ. This can reduce misperceptions and misjudgments. With effective informational exchange between parents and teachers, it should be possible to adjust an educational strategy to a child’s individual needs in order to take the strengths and weaknesses of a child into account.

In order to interpret the results presented here correctly, a few issues need to be taken into consideration. First of all, this large experimental study was performed at four primary schools drawn from the same pool of schools in order to reduce possible differences in background because of regional geography or educational philosophy. Within these four schools, children were selected from low, medium and high SES families. Children from the four grades were balanced with respect to sex and LPE. The sample was homogenized with respect to confounding variables such as repeating or skipping a grade. The choice to control SES and to include only regular students was made in order to reduce variance caused by age and SES of the school. This allowed us to focus evaluation on the core factors sex, LPE, and level of executive functioning as perceived by teachers and parents.

Secondly, we need to consider whether imputing the mean LPE for 27 children by using the mean LPE of a grade from the school of the child affected our results. This has the benefit of not changing the sample mean LPE. On the other hand, it reduces the variability of the data. Thus, this technique could possibly attenuate the standard deviation and the variance (Enders, 2010). However, *post hoc* analyses with exclusion of the children with missing data on the LPE revealed that it had no consequences on the results.

Finally, we need to take into account that although evaluations of teachers and parents were significantly different, the mean differences between both were relatively small and the standard deviation was relatively large. The small differences in means can be explained by the fact that analyses were performed using the mean scores of a relatively large sample size. Differences in evaluations between teachers and parents of an individual pupil might be much greater, but these means were attenuated toward the sample mean. Despite the relatively small difference in mean between parents and teachers, the reported significant difference is highly valuable. The large standard deviation is due to the large variance in teachers’ evaluations of EFs caused by age differences within the sample (children from grade 3 receive lower scores than children from grade 6). The large standard deviation indicates that the AEFI is able to differentiate between children based on their age group.

### Implications

The results of our study provide us with insight into the development of EFs as evaluated by teachers and parents in relation to individual differences at the end of primary school, and a better understanding of the influence of child-related factors on this development. These insights could help us develop successful interventions aimed at improving academic success.

First of all, monitoring and detecting the development of EFs at an early stage (and possible problems in this development) could prove important when it comes to improving study success and to specifying the guidance needed to stimulate an optimal development of these functions. Many researchers have found that EFs were predictive for academic achievements in general (Best et al., 2011; Diamond, 2013; Kautz et al., 2014; Baars et al., 2015). The simple fact that many young adolescents lack adequate skills in planning and self-control could negatively affect their study progress and slow them down in spite of their intellectual abilities (Lowe and Cook, 2003; Titz and Karbach, 2014; Baars et al., 2015). By introducing a new assessment tool, the AEFI, this study tested an instrument that may hold some promise for use in schools in grades 3–6. As our study shows, the AEFI can be used by teachers and parents to monitor the progress of EFs as perceived by teachers and parents in individual children. Hence, it may also be of use in the development of interventions aimed at improving academic success. The relative brevity of the AEFI questionnaire (13 questions) may give it an advantage over other existing questionnaires currently used to evaluate EFs during primary school, such as the widely used BRIEF. The latter instrument contains a substantially larger number of items (namely 86 items), resulting in longer administration times.

Secondly, the insights that this study provides into the relation of the development of EFs as evaluated by teachers and parents to child-related factors such as the sex of the child and LPE could prove important to the success of interventions. In recent years, the amount of research focusing on sex differences has increased rapidly (Lenroot and Giedd, 2010; Dekker et al., 2013; Diamond, 2013; Hyde, 2014; Miller and Halpern, 2014; Rindermann and Baumeister, 2015). This study provides evidence that during young adolescence, boys require specific attention and possibly dedicated educational/pedagogical interventions. This is in line with the results of previous work on the differences in the development of EFs between boys and girls attending high school (Dekker et al., 2013; Vecchione et al., 2014; Baars et al., 2015; Dekker and Jolles, 2015).

Thirdly, this study showed that there are clear differences in evaluations between teachers and parents with regard to girls’ behavior. Helping parents and teachers understand existing differences in their evaluations and why these differences exist could contribute to better collaboration and more openness toward each other. Exchange of information between teachers and parents about a child’s functioning is important because they differ in their perspectives, goals and information about the child. Hence, our study suggests that there is need for improvement of communication between teachers and parents in order to determine the best educational strategy for a child.

## Conclusion

In conclusion, the findings of our study indicate that the development of EFs as perceived and evaluated by teachers and parents during the ages 9–12 years is influenced by the sex of the child as well as by LPE. Moreover, our study shows that when it comes to evaluating the behavior of girls, the evaluations of parents and teachers clearly differ.

This study used an important new assessment tool, the AEFI. This tool can be used by both teachers and parents and provides the means to evaluate how EFs develop with age. Furthermore, it offers important insights into the influence of child-related factors on the development of EFs as perceived and evaluated by parents and teachers in the transition from childhood to adolescence. As such, it could prove an effective instrument in the development of successful interventions aimed at improving academic success.

## Ethics Statement

The study was approved by the Ethics Committee of the Faculty of Movement and Behavioral Sciences of the Vrije Universiteit Amsterdam.

## Author Contributors

MvT: Substantial contributions to the conception and design of the work; the analysis, and interpretation of data for the work; drafting the work; final approval of the version to be published; agreement to be accountable for all aspects of the work in ensuring that questions related to the accuracy or integrity of any part of the work are appropriately investigated and resolved. JJ: Substantial contributions to the conception or design of the work; the acquisition and interpretation of data for the work; revising the work critically for important intellectual content; final approval of the version to be published; agreement to be accountable for all aspects of the work in ensuring that questions related to the accuracy or integrity of any part of the work are appropriately investigated and resolved.

## Conflict of Interest Statement

The authors declare that the research was conducted in the absence of any commercial or financial relationships that could be construed as a potential conflict of interest.
