# Supplementary material for: Teacher Evaluations of Executive Functioning in Schoolchildren Aged 9–12 and the Influence of Age, Sex, Level of Parental Education
Source: Front Psychol. 2017 Apr 3;8:481. doi: 10.3389/fpsyg.2017.00481 (PMC5376587; doi:10.3389/fpsyg.2017.00481)
Supplement: Supplementary file 1 [file Data_Sheet_1.docx]

# Appendix

**Table 5.** Corrected item–scale correlation for the scales of the Amsterdam Executive Functioning Inventory for teachers and parents

*Teachers Parents*

*AEFI Item* Cronbach’s Alpha r_is_ Cronbach’s Alpha r_is_

Attention 0.80 0.59

5 He/she is not able to focus on the 0.63 0.41

same topic for a long period of time

9 He/she is easily distracted 0.70 0.46

12 His/Her thoughts easily wander 0.61 0.33

Self-Control and Self-Monitoring 0.74 0.65

1 He/she often reacts too fast. He/she has 0.32 0.48

done or said something before it is

his/her turn.

7 He/she often loses things 0.66 0.45

8 It takes a lot of time for him/her to finish tasks 0.18 0.36

11 He/she often forgets what he/she has done 0.45 0.35

yesterday

13 It is difficult for him/her to sit still 0.63 0.50

Planning and Initiative Taking 0.81 0.60

2 It is easy for him/her to come up with a 0.66 0.50

different solution if he/she gets stuck when

solving a problem.

3 He/she is well-organized. For example, he/she 0.58 0.41

is good at planning what he/she needs to do

during a day.

4 He/she is full of new ideas 0.55 0.37

6 He/she is curious, he/she wants to know how 0.55 0.24

things work

10 He/she can make fast decisions (e.g., in lesson) 0.63 0.28

*Note.* AEFI = Amsterdam Executive Function Inventory; *r*is = corrected item–scale correlation.
